# Gut microbiota’s causative relationship with peripheral artery disease: a Mendelian randomization study

**DOI:** 10.3389/fmicb.2024.1340262

**Published:** 2024-03-05

**Authors:** Yu Tian, Guanqun Yao, Loren Skudder-Hill, Guangyang Xu, Yuxuan Qian, Feng Tang, Qian Wang, Qianhui Bao, Lei Li

**Affiliations:** ^1^Vascular Surgery Department, Shanxi Bethune Hospital, Shanxi Academy of Medical Sciences, Tongji Shanxi Hospital, Third Hospital of Shanxi Medical University, Taiyuan, China; ^2^School of Clinical Medicine, Tsinghua University, Beijing, China; ^3^Vascular Department, Beijing Hua Xin Hospital (1st Hospital of Tsinghua University), Beijing, China

**Keywords:** atherosclerosis, gut microbiota, Mendelian randomization, metabolites of microbiota, peripheral artery disease

## Abstract

**Introduction:**

The relationship between gut microbiota and peripheral artery disease (PAD) remains understudied. While traditional risk factors like smoking and hyperlipidemia are well-understood, our study aims to determine the potential causative association of gut microbiota with PAD using Mendelian Randomization.

**Methods:**

Data from the International MiBioGen Consortium and the FinnGen research project were used to study 211 bacterial taxa. Instrumental variables, comprising 2079 SNPs, were selected based on significance levels and linkage disequilibrium. Analyses were conducted utilizing the inverse-variance weighted (IVW) method and other statistical MR techniques to mitigate biases, processed in R (v4.3.1) with the TwosampleMR package.

**Results:**

Three bacterial taxa, namely genus *Coprococcus2*, *RuminococcaceaeUCG004*, and *RuminococcaceaeUCG010*, emerged as protective factors against PAD. In contrast, family. FamilyXI and the genus *Lachnoclostridium* and *LachnospiraceaeUCG001* were identified as risk factors.

**Conclusion:**

Our findings hint at a causative association between certain gut microbiota and PAD, introducing new avenues for understanding PAD’s etiology and developing effective treatments. The observed associations now warrant further validation in varied populations and detailed exploration at finer taxonomic levels.

## 1 Introduction

With a staggering public health burden involving over 230 million adults worldwide, peripheral artery disease (PAD) stands as a common and severe manifestation of systemic atherosclerosis (Criqui et al., 2021). Data collected between 2000 and 2010 have indicated that the prevalence of PAD in high-income countries can range from 5.3% in the 45–49 age bracket to up to 18.5% in the 85–89 age group (Fowkes et al., 2013). A comprehensive study in the United States spanning from 1999 to 2004 identified approximately 7.1 million community dwellers with diagnosable PAD (Pande et al., 2011). With the aging global population and subsequent increasing prevalence of risk factors such as hypertension, hyperlipidemia, diabetes, and smoking, the worldwide prevalence of PAD has been on a steady rise. The severity of the health threat attributable to PAD was underscored in 2010 when PAD-associated mortality rates were identified as 31.7, 15.1, and 3.7 years per 100,000 inhabitants in Western, Central, and Eastern Europe, respectively (Hooi et al., 2001). Between the years 1990 to 2010 the PAD-linked mortality rate escalated sharply, peaking at 3.5 per 100,000 individuals in Western Europe by 2010 (Sampson et al., 2014).

The symbiotic relationship between humans and their resident gut microbiota is increasingly recognized as a pivotal determinant of health and disease. Recent years have witnessed a burgeoning body of research establishing an association between the composition and structure of the gut microbial community and atherosclerotic conditions. A 2021 study of 64 participants pinpointed an excess of inflammation-associated gut microbes, like Acidaminococcus, in patients with carotid atherosclerosis, whereas beneficial bacteria, such as anaerobes and butyrate-producing bacteria like Clostridium XVIII/XlVa/XlVb, prevailed at elevated levels in healthy individuals (Ji et al., 2021). An experimental model employing mice on a high-fat diet unveiled that modulating the gut flora, by upregulating Bacteroides and downregulating Bacillota, could ameliorate lipid metabolism and attenuate atherosclerosis (Zhang et al., 2021). Beyond the direct involvement of the gut microbiota in atherosclerotic conditions, metabolites originating from them, like trimethylamine (TMA)—a byproduct of the microbiota’s biosynthesis of phosphatidylcholine in red meat and shellfish, which subsequently gets converted in the liver to trimethylamine N-oxide (TMAO)—have been implicated in atherosclerosis. Elevated plasma TMAO levels have been consistently associated with heightened cardiovascular disease risk, as confirmed by multiple studies (Huang et al., 2023; Molinaro et al., 2023; Shanmugham et al., 2023). Interventional studies involving dietary choline or TMAO supplementation have shown exacerbation of atherosclerotic plaque lesions, with circulating TMAO levels correlating positively with increased intima-media thickness in human carotid arteries.

Although epidemiological insights and related research have furnished evidence pointing towards potential shifts in gut microbiota being implicated in atherosclerotic progression (Hemmati et al., 2023), the precise characteristics of the gut microbiota in PAD patients remain to be elucidated. To address this paucity, the present study capitalized on the Mendelian Randomization (MR) approach, and utilizing summary statistics from genome-wide association studies (GWAS) sourced from the MiBioGen and FinnGen consortiums, aimed to evaluate the potential relationship between gut microbiota and PAD.

## 2 Method

This study is reported following the Strengthening the Reporting of Observational Studies in Epidemiology Using Mendelian Randomization guidelines (STROBE-MR, S1 Checklist).

### 2.1 Data sources

The present study obtained data from the publicly available datasets of GWAS. Specifically, the gut microbiota GWAS data was sourced from the International MiBioGen Consortium (Kurilshikov et al., 2021), accessible at www.mibiogen.org, and incorporates information from 24 cohorts with a total of 18,340 individuals. The GWAS summary statistics for PAD were derived from the FinnGen research project (Kurki et al., 2023), which encompasses an entirely European population.^1^ All original data obtained and used in the present study received relevant ethical approval and informed consent. After adjusting for age, gender, genetic relatedness, genotyping batch, and the first 10 principal components, the analyses of the present study were conducted on 7,098 cases, 206,541 controls, and a total of 16,380,453 SNPs.

### 2.2 Selection of instrumental variables

We began our MR analysis with an initial evaluation of 211 microbial taxa. From this, 15 taxa classified under “unclassified groups” were removed, resulting in a total of 196 distinct bacterial taxa in the analysis. These comprised 9 phyla, 16 classes, 20 orders, 32 families, and 119 genera. Single Nucleotide Polymorphisms (SNPs) related to the gut microbiome were pinpointed and subsequently employed as instrumental variables (IVs). To ensure the authenticity and precision of the inferred causality between gut microbiota composition and PAD, strict criteria were used to select IVs. Firstly, we prioritized SNPs with phenotypic associations at *p* < 1 × 10^−5^ (Long et al., 2023). This threshold was chosen as GWAS-identified loci for gut microbiota seldom reach the conventional genome-wide significance level (*p* < 5 × 10^−8^), allowing us to include a broader spectrum of genetic variations potentially relevant to the associations studied. Second, utilizing the 1,000 Genomes project’s European samples as our reference panel, we computed the linkage disequilibrium (LD) amongst the identified SNPs. Only the SNPs with an *R*^2^ < 0.001 within a clumping window size of 10,000 kb and exhibiting the smallest *p*-values were retained. Third, SNPs presenting a minor allele frequency (MAF) of 0.01 or less were systematically removed from our analyses. Fourth, in cases where palindromic SNPs were detected, we inferred the alleles on the forward strand based on allele frequency data. Fifth, the robustness of each SNP was evaluated via the *F*-statistic. $F=\frac{R^{2}}{1-R^{2}}.\frac{N-K-1}{K}$, where *R*^2^ represented the fraction of the variance in the exposure that was accounted for by the genetic variants, *N* represented the size of the sample, and *K* represented the count of instrumental variables. SNPs with an *F*-statistic ≥10 were deemed strong. Conversely, those with an *F*-statistic below 10 were categorized as weak and excluded from our analyses. Additionally, to further ensure the quality of our selected SNPs, we utilized the PhenoScanner V2 tool available at http://www.phenoscanner.medschl.cam.ac.uk (Kamat et al., 2019) to authenticate the extracted SNPs and eliminate potential confounding SNPs that might have a causal relationship with PAD.

### 2.3 Statistical analysis

#### 2.3.1 Mendelian randomization analysis

In this study, we sought to elucidate the causal relationship between gut microbiota and PAD utilizing well-validated MR methodologies, including the inverse-variance weighted (IVW) test (Zhang et al., 2023), weighted mode (Hartwig et al., 2017), MR-Egger regression (Bowden et al., 2015), weighted median estimator (WME) (Bowden et al., 2016), and simple mode. The backbone of our results, when dealing with multiple instrumental variables, was shaped by the IVW method (Bowden et al., 2016). The additional methodologies provided depth and breadth, ensuring a comprehensive evaluation of our hypothesis.

#### 2.3.2 Sensitivity analysis

Considering that biases might be caused by IVW, sensitivity analyses, including the heterogeneity test, pleiotropy test, and leave-one-out sensitivity test, were also performed to assess the reliability and the stability of MR results. Within the framework of a two-sample MR, we addressed instrument heterogeneity by calculating Cochran’s *Q* statistics. A significant *Q* statistic, emerging either from a value surpassing the count of instruments reduced by one or a *p* < 0.05, signaled potential instrument heterogeneity. Addressing such heterogeneity was vital to our study as it helped ensure the reliability of the MR estimates, preventing potential biases. In attempt to detect horizontal pleiotropy—a potential bias in MR studies—we utilized the MR-PRESSO method (Verbanck et al., 2018). This approach juxtaposed the observed distances of SNPs to the regression line with distances anticipated under a null hypothesis devoid of horizontal pleiotropy. Whenever the data hinted at the presence of horizontal pleiotropy, we substituted outlier-corrected estimates. To further establish the reliability of our conclusions, leave-one-out sensitivity analyses was executed. This allowed us to discern if the causal association was disproportionately influenced by a specific SNP.

**Figure 1:**
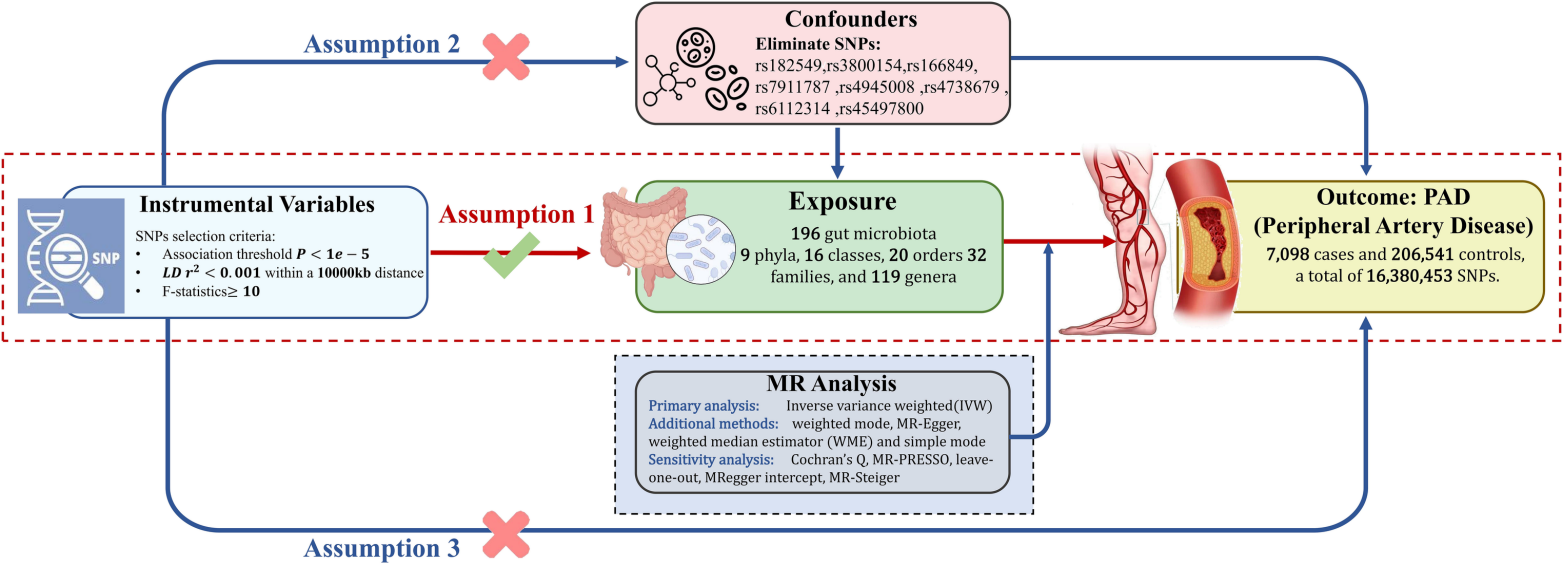
This schematic delineates the Mendelian randomization (MR) analysis framework to investigate the causal link between gut microbiota and Peripheral Artery Disease (PAD). The analysis is predicated on three foundational assumptions: first, there is a significant linkage between selected genetic variants and gut microbiota composition; second, to mitigate confounding effects, SNPs with known confounders were meticulously excluded, based on comprehensive literature review; and third, the genetic variants influence PAD outcomes solely through their impact on gut microbiota. SNPs, single nucleotide polymorphisms; LD, linkage disequilibrium.

The study design flowchart is shown in Figure 1. All computational tasks were conducted using R version 4.3.1. (R Foundation for Statistical Computing, Vienna, Austria). The MR analyses were supported using the TwosampleMR package (version 0.5.7).

## 3 Results

### 3.1 Summary

In this study, through MR analysis, we revealed potential causal links between specific components of the gut microbiota and the risk of developing PAD. We found that certain components of the microbiota may have a protective effect in reducing the risk of PAD, while others could potentially increase the risk of developing the disease. These findings deepen our understanding of how the gut microbiome may influence the risk of PAD.

### 3.2 Identification of potential causal microbial genera

Utilizing the IVW method, nine bacterial taxa exhibited significant associations. These included: class. Actinobacteria, family. Acidaminococcaceae, family. FamilyXI, genus. *Coprococcus2*, genus. *Lachnoclostridium*, genus.*Lachnospirace-aeUCG001*, genus.*RuminococcaceaeUCG004*, genus.*RuminococcaceaeUCG010*, and order.NB1n. Through conducting a comprehensive epidemiological literature survey, specific SNPs were identified to have potential confounders. For instance, the SNPs rs182549 of class. Actinobacteria, rs3800154 of genus.*RuminococcaceaeUCG004*, and rs166849 and rs7911787 of order.NB1n are known to be associated with Body Mass Index (BMI) and weight (Criqui et al., 2021; Lempesis et al., 2023). The SNP rs4945008 of class. Actinobacteria is linked with 25 hydroxy vitamin D concentrations (Norman and Powell, 2005), while rs4738679 of genus. *Lachnoclostridium* exhibits correlation with low-density lipoprotein, total cholesterol, and treatment with atorvastatin (Criqui et al., 2021). Furthermore, rs6112314 of genus. *Lachnoclostridium* has connection to mean platelet volume (Berger et al., 2010), and rs45497800 of family. Acidaminococcaceae shows association with current smoking status (Criqui et al., 2021). All information on SNPs with potential confounding effects can be found in the Supplementary Table S1. A total of 2,175 SNPs were utilized as IVs for 196 unique bacterial genera, as detailed in the Supplementary Table S2.

### 3.3 Main Mendelian randomization results

**Figure 2:**
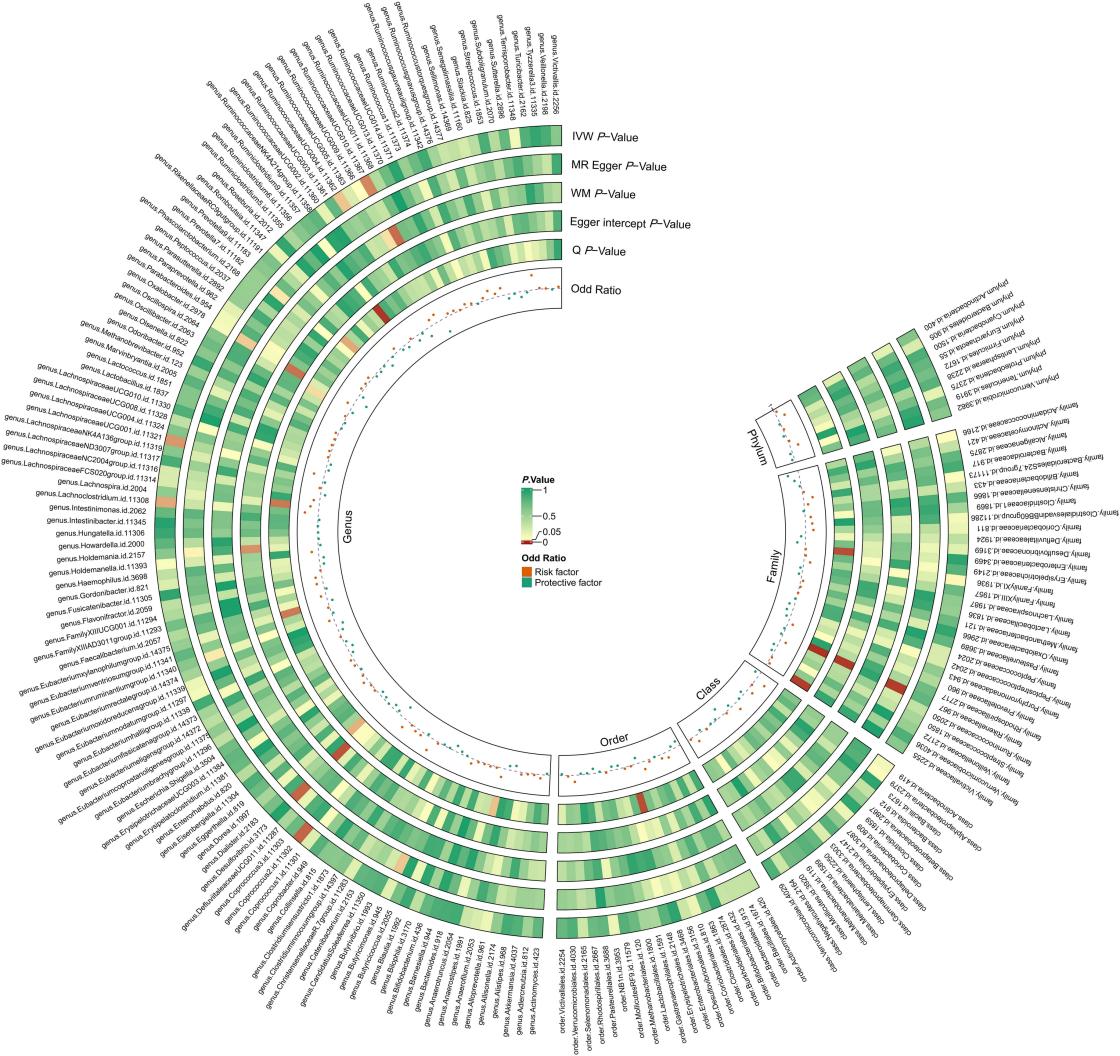
The circus plot presents the causal estimates and sensitivity analyses between gut microbiota and PAD for the different MR methods. IVW *p*-value, the *p*-value of the inverse-variance weighted test; MR Egger *p*-value, the *p*-value of the MR-Egger regression model; WM *p*-value, the *p*-value of the weighted median model; Egger Intercept *p*-value, the *p*-value of Egger’s regression test; *Q p*-value, the *p*-value of Cochran’s *Q* statistic; odds ratio, the odds ratio from the inverse-variance weighted test.

**Figure 3:**
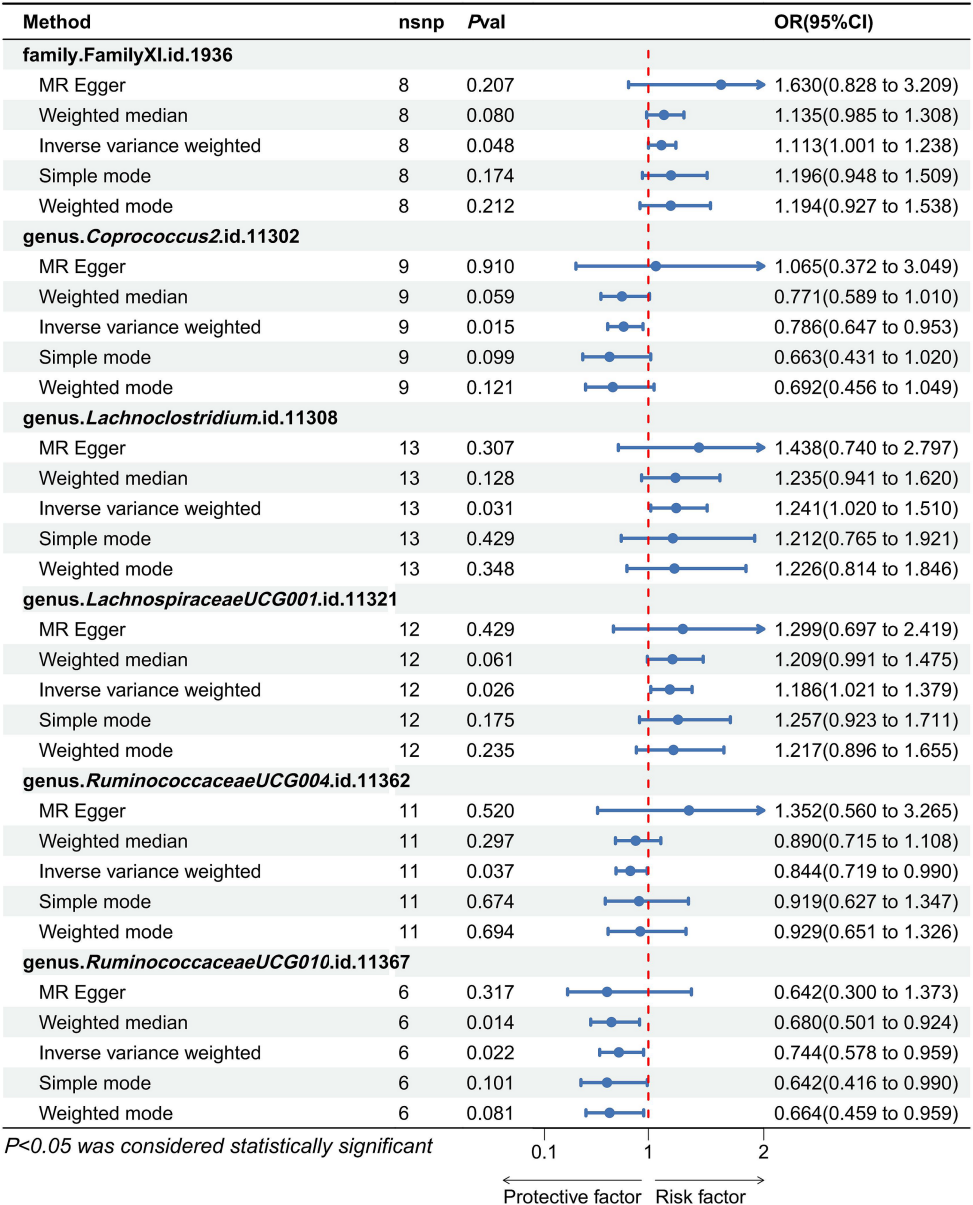
Forest plots show the effect size of each gut microbiota taxa on the pathogenesis of PAD. An odds ratio <1 indicated a protective causal effect, while a ratio >1 indicated a pathogenic causal effect.

The definitive MR results are visually illustrated in Figures 2, 3. A cumulative assessment of these findings is further presented in the Supplementary Tables S3, S4. From the IVW analyses, Genus.*Coprococcus2* (OR = 0.786, 95% CI: 0.647–0.953, *p* = 0.014), Genus.*RuminococcaceaeUCG004* (OR = 0.844, 95% CI: 0.719–0.990, *p* = 0.037), and Genus.*RuminococcaceaeUCG010* (OR = 0.744, 95% CI: 0.578–0.959, *p* = 0.02) were found to act as protective factors against PAD. Conversely, Family.FamilyXI (OR = 1.113, 95% CI: 1.001–1.238, *p* = 0.048), Genus.*Lach-noclostridium* (OR = 1.241, 95% CI: 1.020–1.510, *p* = 0.031), and Genus.*LachnospiraceaeUCG001* (OR = 1.186, 95% CI: 1.021–1.379, *p* = 0.026) were identified as risk factors for PAD. Detailed information on the SNPs for these 6 significant bacterial taxa is available in the Supplementary Table S5.

### 3.4 Examination of outliers and heterogeneity

**Figure 4:**
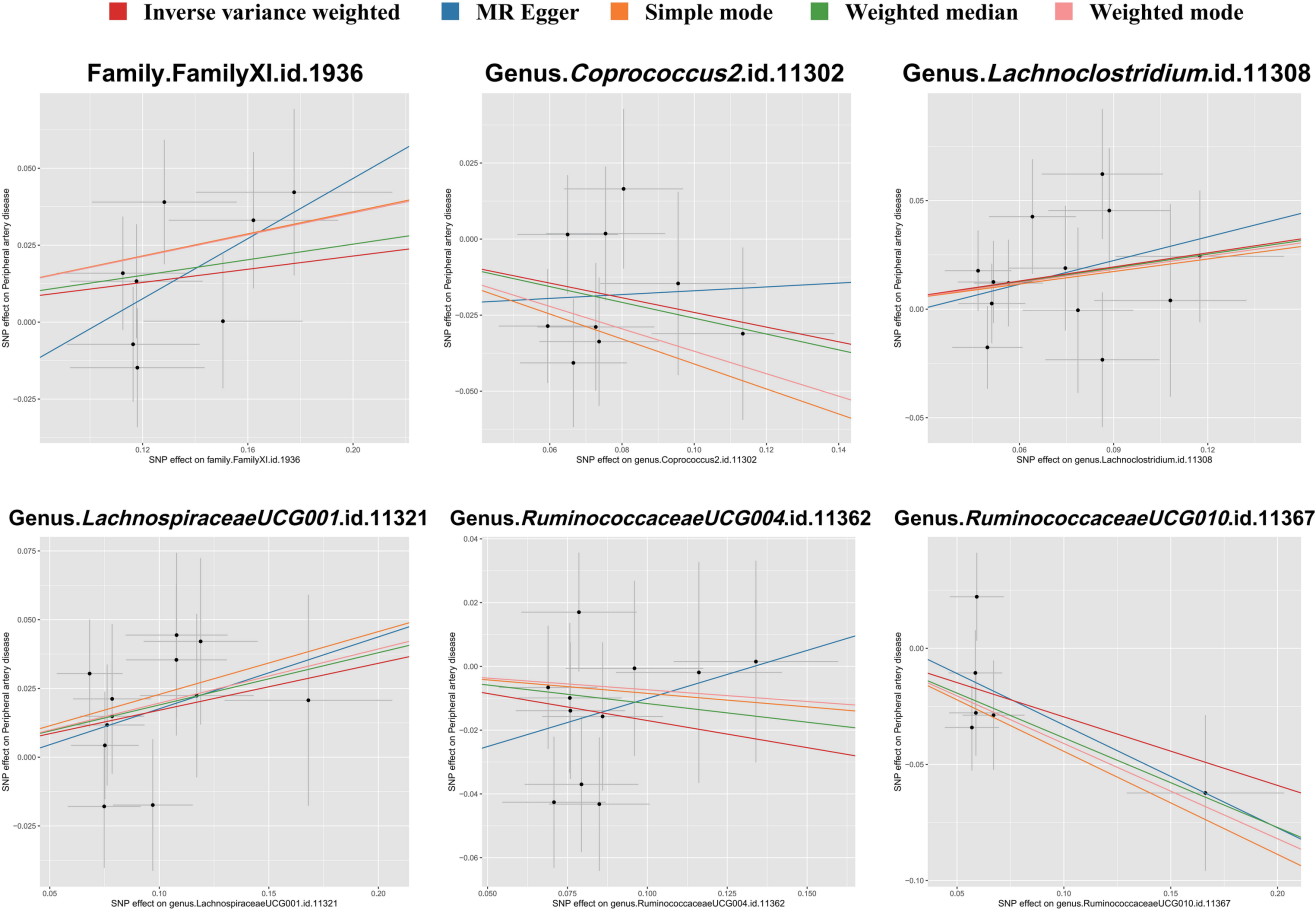
Scatter plots illustrating the associations between gut microbiota taxa and PAD through SNP associations. Each plot shows the correlation of specific taxa-SNP associations (*x*-axis) with PAD-SNP associations (*y*-axis), with 95% confidence intervals denoted by horizontal and vertical lines. Upward sloping lines from left to right indicate a positive correlation, suggesting a potential pathogenic role of the taxa in PAD. Conversely, downward slopes suggest a protective effect. Analyses were conducted using the inverse variance weighted method, weighted mode, MR-Egger regression, weighted median estimator, and simple mode.

**Figure 5:**
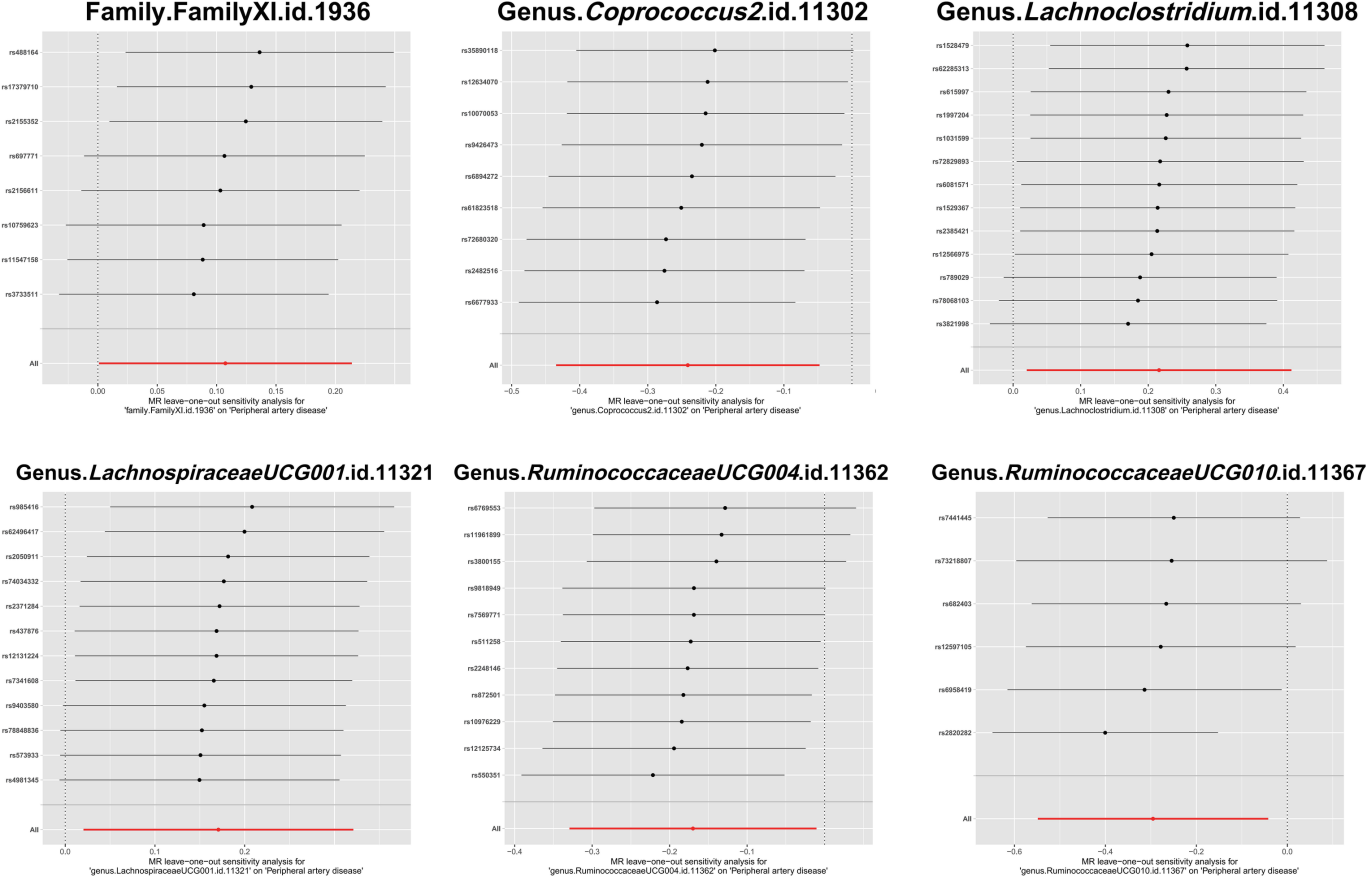
Leave-one-out plots of the causal association between gut microbiota and PAD. These plots depict the results of a leave-one-out sensitivity analysis that was conducted to assess the impact of each individual SNP on the pooled effect size. Each point on the plot represents the recalculated pooled effect size when the corresponding SNP on the *y*-axis is excluded from the analysis. The horizontal lines indicate the 95% confidence intervals of the recalculated effect sizes.

To evaluate heterogeneity and potential outliers in our data, we utilized scatter plots (Figure 4) and leave-one-out plots (Figure 5). While the genus *RuminococcaceaeUCG010* was suspected to harbor potential outliers, no significant heterogeneity was detected from the Cochran’s *Q* test results as shown in Table 1. This was corroborated by the non-significant *p*-values for Family XI (*p* = 0.482), genus *Coprococcus2* (*p* = 0.604), genus *Lachnoclostridium* (*p* = 0.703), genus *LachnospiraceaeUCG001* (*p* = 0.814), genus *RuminococcaceaeUCG004* (*p* = 0.505), and genus *RuminococcaceaeUCG010* (*p* = 0.302), suggesting a lack of heterogeneity across these taxa.

**Table 1:** Supplementary and sensitivity analyses for causality from gut microbiota on peripheral artery disease.

Exposure	Cochran’s *Q*	MRegger_intercept	MRegger_intercept_pval	Steiger_pval	MRpresso_global
family.FamilyXI	0.482	−0.051	0.307	2.68E-39	0.511
genus.Coprococcus2	0.604	−0.023	0.582	2.95E-36	0.644
genus.Lachnoclostridium	0.703	−0.010	0.658	8.23E-51	0.708
genus.LachnospiraceaeUCG001	0.814	−0.009	0.775	3.92E-54	0.819
genus.RuminococcaceaeUCG004	0.505	−0.040	0.315	2.85E-47	0.490
genus.RuminococcaceaeUCG010	0.302	0.011	0.703	4.69E-26	0.385

Table 1 also presents results from the horizontal pleiotropy evaluation and the Steiger test. In the MR-PRESSO global test, the *p*-values for Family XI (*p* = 0.511), genus *Coprococcus2* (*p* = 0.644), genus *Lachnoclostridium* (*p* = 0.708), genus *LachnospiraceaeUCG001* (*p* = 0.819), genus *RuminococcaceaeUCG004* (*p* = 0.490), and genus Ruminococ-caceaeUCG010 (*p* = 0.385) were all insignificant, indicating no significant horizontal pleiotropy. Consistency in results was observed with the MR-Egger regression, which aligned with the findings from the MR-PRESSO global test. Furthermore, the outcomes of the Steiger test, featuring *p* < 0.05, suggested a strong association between the IVs and gut microbiota. These results confirm the reliability of the MR findings, and the correct directionality of the causative association between gut microbiota and peripheral artery disease.

## 4 Discussion

Traditionally, the primary recognized risk factors for PAD have centered on elements such as smoking and hyperlipidemia. Comparatively, few studies have delved into the gut microbiota’s potential role as a risk or protective factor for PAD. Based on our extensive literature review, the present study is the first MR investigation to identify a causative relationship between gut microbiota and PAD. Our investigation assessed the causal effects of potential microbial modulators in PAD patients, unveiling a plausible causative connection between gut microbiota and PAD. Initial results identified significant associations between certain gut microbial groups and PAD, notably class Actinobacteria, family Acidaminococcaceae, and order NB1n. After the exclusion of confounding SNPs, these associations weakened. Nonetheless, the associations for FamilyXI, genus *Coprococcus2*, genus *Lachnoclostridium*, genus *LachnospiraceaeUCG001*, genus *RuminococcaceaeUCG004*, and genus *RuminococcaceaeUCG010* still remained significantly associated with PAD.

Prevailing evidence suggests that the abundance of *Lachnoclostridium* is significantly associated with atherogenesis (Cai et al., 2022). It is also known that L. saccharolyticum WM1 in *Lachnoclostridium* effectively transforms choline into microbial TMA at an almost 100% conversion rate. In the liver, TMA is converted to TMAO (Cai et al., 2022). TMAO is a metabolite of the gut microbiota correlated with atherosclerosis and has been confirmed as a high-risk factor for cardiovascular diseases (Zhao and Wang, 2020; Thomas and Fernandez, 2021; Anto and Blesso, 2022). Animal studies have revealed that supplementing diets with choline or TMAO can exacerbate atherosclerotic plaque lesions (Canyelles et al., 2023). Elevated levels of TMAO can inhibit reverse cholesterol transport and bile acid metabolism, amplifying vascular inflammation and cellular apoptosis, leading to atherosclerosis (Chen et al., 2023). Moreover, other studies have shown the links of *Lachnoclostridium* with conditions like obesity (Guo et al., 2023), hypertension (Maruyama et al., 2023), and diabetes (Zang et al., 2023). Our findings, suggesting an association between *Lachnoclostridium* and PAD, further underscore the role of this bacterial genus in vascular diseases.

*Lachnospiraceae*, a key component of the gut microbiota and a major producer of short-chain fatty acids (SCFAs), generally supports metabolic health (Vacca et al., 2020; Zhang et al., 2023). However, under specific conditions, it might lead to detrimental health outcomes. Research indicates that an increase in *Lachnospiraceae*, linked to consuming a high-fat diet, is closely associated with the onset of metabolic syndrome (Ye et al., 2023), including factors such as central obesity (Witkowski et al., 2020) and insulin resistance (Wolosowicz et al., 2022). These factors are crucial risk elements for the development of Peripheral Artery Disease (PAD). In particular, a high-fat diet-induced increase in *Lachnospiraceae* correlates with alterations in lipid metabolism, which could elevate the risk for PAD. Moreover, a high-fat diet alters not just the gut microbiota’s composition, specifically increasing *Lachnospiraceae* numbers, but also its metabolic activity. This could indirectly influence the progression of PAD by affecting lipid metabolism and inflammatory pathways (Vacca et al., 2020). Thus, while *Lachnospiraceae’s* role in producing SCFAs is beneficial, aiding in the maintenance of both gut and overall metabolic health, its proliferation under a high-fat diet may pose a potential risk for PAD. This hypothesis necessitates further experimental validation.

Existing evidence has shown that *Coprococcus* is associated with the production of secondary bile acids (Witkowski et al., 2020), which can influence lipid metabolism and systemic inflammatory pathways, representing microbiome metabolites with profound health implications. This points to a potential protective mechanism of this bacterial genus in PAD. While some research has suggested an association between specific species of *Ruminococcus* and metabolic dysfunction related to obesity, other studies have highlighted its role in modulating bile acid metabolism (Molinero et al., 2022). Changes in the bile acid profile and their impact on lipid metabolism and liver function might indirectly affect the risk of PAD. In a study focused on dietary modifications for blood glucose regulation (Huang et al., 2020), *Ruminococcaceae* exhibited protective potential. Current research on *Ruminococcaceae* mostly portrays them as protective factors in diseases related to cardiovascular risk factors, but some studies have also hinted at their role as risk factors. This inconsistency may be related to differences in sub-genus categorization. In our study, genus. *Ruminococcaceae* did not reach statistical significance during MR analysis, while genus.*RuminococcusUCG004* and genus.*RuminococcusUCG010* demonstrated statistically significant, protective effects against PAD.

In many observational studies of gut microbiota, discussions related to the functionalities and metabolites of microbiota pertinent to our study largely focus on the genus level. However, the key findings of our study majorly revolve around sub-genus levels. For instance, risk factor genus *LachnospiraceaeUCG001* is a sub-genus of *Lachnospiraceae*, protective factor genus *Coprococcus2* is a sub-genus of *Coprococcus*, and both *Ruminococcaceae-UCG004* and *RuminococcaceaeUCG010* are sub-genera of *Ruminococcaceae*. There are palpable distinctions between each sub-genus and genus, emphasizing the need for more refined and precise gut microbiota research in the future.

Researchers have begun to explore the prevention of atherosclerosis through adjustments in gut microbiota. It has been reported that gut microbiota-driven metabolic byproduct TMAO is a promising core drug target for various conditions, including cardiovascular diseases (Cao et al., 2023). Small molecules inhibiting gut microbial TMA lyases have been proposed as potential therapeutic strategies to mitigate atherosclerosis. A clinical trial of Taurisolo^®^, an innovative nutraceutical formulation derived from grape pomace polyphenols, evidenced its anti-atherosclerotic efficacy by showing reduced circulating TMAO levels (Chen et al., 2023).

Research on *Lachnoclostridium*, which our study has identified as positively associated with PAD, has shown that supplementing with plant Lactobacillus FRT10 can significantly reduce the abundance of *Lachnoclostridium*, which in turn can considerably lower body weight gain and liver concentrations of triglycerides and alanine transaminase (Cai et al., 2020). This provides promising support for that, in the future, controlling gut microbiota may provide new avenues for the treatment and prevention of PAD.

It is crucial to acknowledge that while MR offers a robust framework for inferring causal relationships, it comes with inherent limitations, including population stratification where genetic variants might be distributed unevenly across different ancestries, potentially confounding the associations. Moreover, the human gut contains a complex and diverse symbiotic microbial system. While bacteria are the primary components of the gut microbiome, other symbionts like viruses, fungi, and archaea also play significant roles. Recent studies (Chen et al., 2023; Li et al., 2024) have suggested a link between viruses and the incidence of atherosclerotic diseases, indicating that other components of the human symbiotic microbial system, beyond bacteria, may have associations with PAD that warrant further investigation. Our current analysis focuses solely on the correlation between the gut microbiota and PAD, leaving the relationships between other components of the human symbiotic microbial system and PAD to be explored in future research. This underscores the imperative to validate our study’s findings through further investigations conducted on diverse populations and by employing alternative methodologies to reinforce the robustness of the observed associations. Another limitation of our study pertains to the granularity of the microbiota data. Our discussions on the gut microbiota are primarily constrained to the genus level, a taxonomic rank that groups closely related species based on similar characteristics. Each genus comprises multiple species, which might harbor distinct functionalities and associations with PAD. Thus, future investigations and subsequent clinical translations might benefit from analyzing data at the species level. Such granularity can provide a more comprehensive understanding of the role of the gut microbiota in PAD and its potential as a therapeutic target. Additionally, it is important to note that the data sources we currently use have not efficiently provided specific information about the patient group and the control group, such as disease diagnoses, disease categorization, age, gender, and have not conveniently supplied relevant scores for the gut microbiota. The absence of this information may lead to reader confusion or result in an incomplete study.

## 5 Conclusion

Research on gut microbiota represents a relatively novel frontier, diverging from traditional risk factors for PAD. Leveraging the MR approach, this study identified three genera of gut microbiota as protective factors against PAD, and one family along with two genera as risk factors for PAD. These findings pave the way for subsequent clinical investigations regarding the interplay between gut microbiota and PAD. Future studies are necessary to explore the underlying mechanisms of the associations identified in the present study, and to validate these results across broader and more diverse populations thus ensuring their generalizability and robustness.

### 5.1 Declaration of generative AI and AI-assisted technologies in the writing process

During the preparation of this work the authors used Chat-GPT in order to enhance the written quality of this paper. After using this tool, the authors reviewed and edited the content as needed and take full responsibility for the content of the publication.

## Data availability statement

The original contributions presented in the study are included in the article/Supplementary material, further inquiries can be directed to the corresponding author.

## Ethics statement

The data used in this study were entirely sourced from publicly accessible databases, originally collected by other researchers who had obtained the necessary ethical approvals and informed consent from participants. Furthermore, no individual-level data were utilized in this study. Therefore, no new approval from an ethical review board was required.

## Author contributions

YT: Conceptualization, Data curation, Funding acquisition, Writing – original draft, Writing – review & editing. GY: Data curation, Writing – original draft. LS-H: Writing – original draft. GX: Formal analysis, Writing – review & editing. YQ: Formal analysis, Writing – review & editing. FT: Conceptualization, Writing – review & editing. QW: Formal analysis, Writing – review & editing. QB: Formal analysis, Writing – review & editing. LL: Conceptualization, Methodology, Project administration, Writing – review & editing.

## Footnotes

^1^ https://gw-as.mrcieu.ac.uk/datasets/finn-b-I9_PAD/
